# Chronic Inflammation in Primary Myelofibrosis: In-Depth Insights Into Pathogenesis and Promising Anti-Inflammatory Therapeutic Strategies

**DOI:** 10.1155/mi/9967975

**Published:** 2025-11-12

**Authors:** Meng Chen, Chengyulong Zheng, Ying Zhang, Jiayu He, Zhexin Shi

**Affiliations:** ^1^Department of Hematology, First Teaching Hospital of Tianjin University of Traditional Chinese Medicine, Tianjin, China; ^2^Department of Hematology, National Clinical Research Center for Chinese Medicine Acupuncture and Moxibustion, Tianjin, China

**Keywords:** immune system dysregulation, inflammation, inflammatory mediators, oxidative stress, primary myelofibrosis (PMF)

## Abstract

Primary myelofibrosis (PMF) is a clonal myeloproliferative neoplasm (MPN) stemming from hematopoietic stem and progenitor cells (HSPCs), frequently associated with mutations in Janus kinase signal transducer (JAK2), calreticulin (CALR), or thrombopoietin receptor (MPL). Characterized by intricate clonality and dysregulated inflammation, PMF leads to heightened morbidity, mortality, and an elevated risk of leukemic transformation. The inflammatory state in PMF results from the convoluted interplay of excessive inflammatory mediator production, heightened oxidative stress, and immune system dysregulation. These factors fuel the expansion of the myeloproliferative clone, accelerate disease progression toward leukemia, and contribute to bone marrow (BM) fibrosis by acting on BM stromal cells. This review comprehensively integrates recent research findings, especially those from single-cell RNA sequencing, to identify the sources and regulatory mechanisms of inflammatory mediator overproduction, oxidative stress, and immune system dysregulation in PMF. It also elaborates on the key cytokines and pathways governing the interaction between malignant hematopoietic cells and BM stromal cells. By providing a comprehensive perspective, this review aims to guide future research on cellular and molecular targets within the hematopoietic niche and explore therapeutic approaches targeting immune cells and cytokines for PMF treatment. The potential of anti-inflammatory therapies in the clinical management of PMF is also highlighted.

## 1. Introduction

Primary myelofibrosis (PMF) is a Philadelphia chromosome-negative (Ph –) myeloproliferative neoplasm (MPN) that originates from a clonal myeloproliferation of hematopoietic stem and progenitor cells (HSPCs) [1]. PMF is frequently, though not exclusively, associated with driver genetic mutations in the Janus kinase signal transducer (JAK2) (JAK2V617F) (50%–70%), the chaperone protein calreticulin (CALR) (10%–30%), or the thrombopoietin receptor (MPL) (10%), which either directly or indirectly leads to a deranged Janus kinase (JAK)-signal transducer and activator of transcription (STAT) pathway [1]. The JAK-STAT pathway, an evolutionarily conserved signaling mechanism, is critical for various physiological processes such as hematopoiesis, immune regulation, differentiation, and metabolism [2]. In PMF, constitutive JAK-STAT activation significantly contributes to the proliferation of malignant clones and the exacerbation of inflammatory responses [2]. Recent research utilizing single-cell multiomics has demonstrated that the overproduction of inflammatory mediators not only fuels the proliferation of malignant clones [3] but also accelerates the progression to leukemia [4]. Furthermore, these mediators act on bone marrow (BM) stromal cells, leading to BM fibrosis, increased microvessel density, and osteosclerosis, which in turn disrupt normal hematopoiesis [5]. Moreover, circulating cytokine levels are elevated in PMF patients. Notably, a subset of these cytokines has been identified as poor prognostic indicators [6], highlighting their significance in disease progression and patient outcomes.

As described above, aberrant activation of the JAK-STAT pathway not only drives abnormal cell proliferation but also significantly impacts overall manifestation of the disease. Among Ph–MPN, PMF presents distinct clinical features. It is characterized by extramedullary hematopoiesis, splenomegaly, constitutional symptoms, BM fibrosis, anemia, and an elevated risk of transformation into AML [7]. These clinical features are intrinsically tied to the underlying genetic makeup of PMF. The genetic landscape of PMF is intricate. While the aforementioned driver mutations are common, patients lacking the aforementioned driver genetic mutations are classified as triple-negative PMF. With the advent of high-throughput next-generation sequencing, significant molecular heterogeneity within PMF has been revealed. This has led to the identification of cooperating mutations in high-molecular-risk genes [8], such as TET2, U2AF1, IDH1/2, EZH2, SRSF2, and ASXL1. These mutations promote clonal evolution and disease transformation, accompanied by elevated levels of inflammatory cytokines and alterations in the inflammatory state [9].

The complex genetic milieu in PMF has an immediate influence on the in vivo inflammatory microenvironment. In truth, these clinical manifestations are, at least to some extent, a consequence of the derangement in inflammatory cytokine production [6]. In 2018, Cacemiro et al. [10] conducted an analysis of cytokine profiles across all three subtypes of Ph–MPN, ultimately arriving at the conclusion that PMF has the highest inflammatory burden among them. In fact, an increasing body of evidence emphasizes that chronic inflammation is a crucial driver in the progression of PMF. PMF typically progresses through distinct stages. It starts with the early tumor stage, pre-PMF, which is characterized by the hyperplasia of granulocytic and megakaryocytic lineages and only minimal fibrosis. As the disease advances, it enters the advanced myelofibrotic stage, overt-PMF. If left untreated, this progression may eventually result in transformation into AML. The significance of inflammation in this process is further underscored by research on gene expression patterns. In 2019, Wong et al. demonstrated significant differences in inflammatory gene expression patterns between MPN patients with prefibrotic stage and those with marked fibrosis [11]. Specifically, prefibrotic MPN patients exhibited low cytokine gene expression profiles, while those with marked fibrosis showed upregulation of inflammatory genes [11].

Given the central role of the JAK-STAT pathway in PMF, JAK-STAT inhibitors have emerged as a major treatment approach. However, despite JAK-STAT activation being a key driver in PMF, the complex clonal architecture of the disease, the diverse mutational spectrum, and the presence of coexisting inflammation pose challenges [12]. Consequently, JAK-STAT inhibitors are unlikely to match the impact of imatinib in chronic myeloid leukemia. Ruxolitinib, the first JAK1/2 kinase inhibitor, effectively alleviates splenomegaly and constitutional symptoms. However, it has limited efficacy in reversing BM fibrosis and reducing the burden of mutant gene. The therapeutic benefits of ruxolitinib, attributed in part to its ability to downregulate pro-inflammatory cytokines [13, 14], highlight the importance of targeting inflammation in PMF treatment. Currently, the treatment landscape for PMF is limited. Thus, developing new therapeutic strategies that can target inflammation more effectively and address the root causes of the disease is urgently needed.

Next-generation sequencing has significantly advanced our understanding of the genomics of PMF. Cre-inducible knock-in mouse models have also elucidated the role of collaborative mutations, along with the key driver mutation JAK2V617F, in promoting clonal expansion. However, curative therapies are still lacking, in part because the pathogenesis of BM fibrosis is not well understood. Patients with PMF experience an extended latency before symptom onset postmutation acquisition [15, 16]. This latency disrupts the cellular and stromal interactions within the entire hematopoietic niche in the BM. Accumulating evidence suggests that PMF is not just a disease of HSCPs but a disease of the entire hematopoietic niche in BM [17]. To address this knowledge gap, future research should focus on identifying the pivotal inflammatory mediators and underlying molecular mechanisms that disrupt the intricate crosstalk between HSPCs and the BM hematopoietic microenvironment. Given the wide range of upregulated inflammatory mediators in PMF, it is unlikely that these disruptions are limited to a single pathway. Emerging evidence points to distinct molecular mechanisms controlling BM fibrosis and niche cell fate [18]. For example, inhibiting transforming growth factor (TGF)-β signaling in mesenchymal stem cells (MSCs) halts the progression of BM fibrosis induced by MPLW515L. However, this intervention does not fully restore the compromised hematopoietic niche. This indicates that a more comprehensive understanding of the crosstalk between different cell types and signaling pathways is required to develop more effective therapies.

Single-cell RNA sequencing has emerged as a promising tool in the study of PMF. It provides detailed insights into the transcriptome at the single-cell level. The single-cell resolution allows for the identification of rare cell types, presenting an unprecedented opportunity to uncover cellular and molecular heterogeneity and identify targets specific to malignant hematopoietic cells that were previously difficult to detect [19, 20]. Complementing this, multiomic single-cell methods, capable of simultaneously analyzing multiple data types, offer a high-resolution cellular atlas of myelofibrotic BM. This comprehensive view encompasses both hematopoietic and stromal cell populations. Through such an all-inclusive analysis, we can significantly enhance our understanding of PMF pathogenesis. For example, it can uncover previously unrecognized cell–cell interactions that contribute to inflammatory mediators. Additionally, it can elucidate the mechanisms underlying the abnormal differentiation of fibroblasts, thereby pinpointing their potential origins. The knowledge gleaned from these analyses can be directly translated into the identification of novel cellular and molecular targets for anti-inflammatory therapies. These therapies aim to impede the progression of malignant clones and reverse the transition to BM fibrosis [5]. In this review, we have explored recent findings from single-cell analysis on MPN, with a focus on PMF, highlighting the crucial role of inflammation in PMF pathogenesis and the potential of these new techniques to revolutionize our approach to treating this complex disease.

## 2. Inflammation in PMF: Pathophysiological Insights

In recent years, cancer-related inflammation has been acknowledged as a crucial factor in cancer progression, whether it stems from a chronic inflammatory disease or is induced by a tumor. It facilitates unbridled replicative capacity, autonomy from growth signals, resistance to suppressive cues, avoidance of apoptotic pathways, augmented angiogenesis, tumor dissemination and, eventually, metastasis [21]. This understanding has spurred several studies, indicating that specific anti-inflammatory drugs have significant potential in modulating the tumor immune environment, capitalizing on their anti-inflammatory properties [22].

Over the past decade, the hypothesis that MPN is chronic tumor models propelled by inflammation has been proposed [23, 24]. A multitude of studies subsequent to the proposal of this hypothesis have provided substantial evidence to support this. Prior to delving deeper into the role of inflammation in MPN, it is imperative to introduce clonal hematopoiesis of indeterminate potential (CHIP). CHIP, characterized by somatic mutations in hematopoietic stem cells (HSCs), results in clonal expansion of one or more hematopoietic lineages in individuals without overt hematological malignancies. Emerging evidence indicates CHIP as a significant precursor in the development of hematological disorders, MPN included. Among these mutations, JAK2 mutations are more frequently detected in clonal hematopoiesis compared to CALR or MPL mutations. These JAK2 mutations promote the expansion of myeloid cells and can lead to the development of MPN [25]. In a conditional knock-in mouse model of JAK2V617F, JAK2V617F was found to impair the function of HSCs. Moreover, the accumulation of DNA damage promotes clonal expansion and disease progression [26]. Notably, pro-inflammatory cytokines are pivotal in the transition of CHIP to MPN. A recent study developed an experimental system employing competitive BM transplantations at high dilutions, with each recipient receiving only 1–3 HSCs. The study revealed that the absence of interleukin (IL)-1β in JAK2-mutant HSCs led to reduced engraftment, constrained clonal expansion, a decrease in the total number of functional HSCs, and a lower rate of progression to MPN [27]. This research not only highlights the pivotal role of IL-1β-mediated inflammation in facilitating the transition of JAK2V617F-mutated clonal hematopoiesis to MPN but also underscores the potential of targeting IL-1β during the extended preclinical phase of clonal expansion to prevent or delay malignant transformation. By intervening at this stage, we may be able to prevent or delay the malignant transformation that leads to the development of PMF.

As MPN, especially PMF, predominantly affects the elderly, the impact of aging on the disease mechanism cannot be overlooked. Aging is intrinsically linked to a rapid decrease of clonal diversity [28], alongside an elevation in inflammatory mediators [29] and a diminished capacity of inflammatory signals to regulate tissue homeostasis and facilitate tissue regeneration. In the BM, the concentrations of key inflammatory cytokines, such as tumor necrosis factor (TNF) and interferon (IFN)-γ, increase with age. This increase skews cell differentiation toward a myelomonocytic lineage [30]. Pang et al. [31], by observing HSPCs in senescent rats, reported that senescence is accompanied by a decrease in lymphocyte production and a myeloid bias, with the immune milieu being remodeled by the accumulation of senescent inflammatory cells. There is increasing evidence supporting that the blood stem cells age differently in humans and mice, hence warranting for attention when interpreting results across species [32]. Both age and comorbidities are recognized as contributing factors to immune senescence [33], a state characterized by the dysfunction and decline of immune responses. PMF patients, who are mostly elderly and often have comorbidities, experience a notable latency period between mutation acquisition and the onset of clinical symptoms. This latency period serves as a window into the complex interplay between genetic mutations and the subsequent development of the disease. In the context of PMF, chronic inflammation, which is often more pronounced in the elderly, plays a crucial role. When HSPCs acquire driver gene mutations, chronic inflammation confers a proliferative advantage. It also enables these cells to evade immune surveillance. This dual effect leads to the gradual expansion of malignant clones, eventually resulting in the clinical manifestations and disease progression characteristic of PMF.

Gene expression profiling studies [32] have revealed that patients with PMF experience extensive deregulation of numerous genes involved in inflammatory responses, oxidative stress, and immune modulation. These factors intricately interact and are deeply intertwined in the pathogenesis of PMF. The myeloproliferative clone in PMF, predominantly composed of monocytes and megakaryocytes, is a constant source of inflammatory mediators. Pro-inflammatory cytokines such as TNF [3] and IL-1 [34] are key players. They play crucial roles in maintaining and expanding the myeloproliferative clone through autocrine or paracrine mechanisms [3]. A recent study utilizing single-cell multiomics identified TP53-mutant HSPCs with dysregulated inflammation-associated gene expression. These mutant HSPCs are enriched in MPN patients who are prone to develop TP53-mutant secondary AML. In vivo experiments further demonstrated that inflammation stimulates the genetic evolution of Trp53-mutant mouse HSPCs, highlighting chronic inflammation as a significant driving force in the progression of TP53-mutant leukemic evolution [4]. In addition to the overproduction of pro-inflammatory cytokines, PMF-associated driver mutations also trigger oxidative stress in the malignant clone [35, 36], leading to oxidative DNA damage and DNA replication stress. Furthermore, PMF-associated driver mutations also enhance the expression of programmed death (PD) ligand-1(PD-L1) [36]. PD-L1 significantly impairs the function of wild-type T cells, triggers immune dysregulation, and enables the malignant cells to evade both local and systemic immune control by the host. Consequently, this accelerates the progression of the disease.

JAK-STAT inhibitors, such as ruxolitinib, are successful in reducing cytokine levels. However, they are ineffective in managing oxidative stress and immune dysregulation. Evidence shows that they have no influence on H_2_O_2_ onset or the overall level of DNA oxidative modifications [37], emphasizing the urgent need for new therapies to address these fundamental challenges in PMF treatment. Exploring their cellular and molecular mechanisms of chronic inflammation in PMF can not only deepen our understanding of PMF but also help identify novel therapeutic targets, thus laying a solid foundation for enhancing patient treatment. The subsequent section will delve into the cellular and molecular mechanisms responsible for the overproduction of inflammatory mediators.

## 3. Cellular and Molecular Mechanism of Inflammatory Mediator Overproduction

### 3.1. Pro-Inflammatory Signaling Pathways in PMF

#### 3.1.1. JAK-STAT Signaling in PMF

Uddalak et al. [38] reported that JAK-STAT signaling exerts a major influence on inflammation generation, modulation of the immune environment, and development of fibrosis. The JAK-STAT signaling cascade, once activated by driver gene mutations, plays a pivotal role in facilitating the production of a variety of inflammatory mediators, including TNF. TNF, as a cytokine central to modulate inflammatory responses, is implicated in a wide spectrum of inflammatory and autoimmune diseases [39]. It serves as a key mediator that amplifies the inflammatory process. Studies by Tyner et al. utilizing murine models revealed elevated TNF levels in PMF [40]. Subsequent cell-based assays further revealed that the JAK2V617F mutation regulates TNF expression in both primitive hematopoietic cells and cell lines harboring JAK2V617F, ultimately enhancing the physiological JAK2-dependent TNF production [3]. Hyperactivated JAK-STAT signaling leads to the production of a variety of inflammatory mediators regardless of the mutation type in MPN. Despite this, JAK-STAT signaling is not the sole source of cytokines in PMF, as JAK2 inhibition alone does not normalize cytokine levels [41].

#### 3.1.2. NF-κB Signaling in PMF

Growing evidence suggests that nuclear factor kappa-light-chain-enhancer of activated B cells (NF-*κ*B) is a key transcriptional coregulator driving aberrant inflammation, which, together with activated STAT3, contributes to distinct pathologies [42]. NF-*κ*B is a family of inducible transcription factors that respond to a wide range of stimuli. Abnormalities in NF-*κ*B function have been implicated in various inflammatory disorders, which encompass rheumatoid arthritis, inflammatory bowel diseases, and the pathological processes underlying atherosclerosis [43]. Most NF-*κ*B target genes are overexpressed in CD34^+^ cells from PMF patients [44]. In PMF, patients with JAK2 mutations display increased expression levels of NF-*κ*B pathway target genes compared to those without JAK2 mutations in PMF [45]. Indeed, the NF-*κ*B signaling pathway is overactivated in both malignant and nonmalignant cells [44, 46]. This hyperactivation is triggered by both cell-autonomous signaling from mutant JAK2 kinase and noncell autonomous activation by inflammatory mediators, with TNF playing a significant role [44].

#### 3.1.3. The Cooperation Between NF-κB Pathways and STAT3 in PMF

The cooperation between NF-*κ*B pathways and STAT3 in regulating key target genes drives the inflammatory state in PMF. Inhibiting the JAK pathway, coupled with the indirect inhibition of NF-*κ*B pathway using the bromodomain and extraterminal (BET) inhibitor JQ1, has been effective in reversing BM fibrosis [46]. In miR-146a-deficient mice, the myelofibrosis (MF)-like phenotype was induced by the deletion of miR-146a not by mutations in driver genes [47]. Moreover, the NF-*κ*B inhibitor BMS-345541, targeting IKKα/β, has been shown to reduce extramedullary hematopoiesis, BM fibrosis, and osteosclerosis and to alleviate inflammation, as indicated by decreased levels of IL-1β and TNF [47]. In patient-derived xenograft mouse models, the abrogation of NF-*κ*B cascade effectors, including RelA, Myd88, and IL-1 receptor-associated kinase (IRAK) 4, consistently led to the suppression of leukocytosis, splenomegaly, and BM dysfunction [48]. These findings indicate that the NF-*κ*B pathway is a key driver of PMF progression and a viable target for therapeutic intervention. Pelabresib (CPI-0610), a selective oral BET domain inhibitor, has shown promising results in combination with ruxolitinib in JAK inhibitor–naïve MF, with durable improvements in spleen and symptom response, and good tolerability [49].

#### 3.1.4. The Role of Collaborative Genetic Mutations in PMF

Beyond these well-studied pathways, collaborative genetic mutations involved in epigenetic regulation, RNA splicing, and cellular signaling play a key role in the production of cytokines and chemokines, either independently or in combination with driver mutations. Based on epigenetic studies, IDH1/2 and TET2 mutations result in downregulation of IL-11 receptor α and TGF-β receptor 1 expression, thereby reducing the suppressive effect of TGF-β on clonal HSPCs and the anti-inflammatory actions of IL-11 [50]. U2AF1 mutations have been implicated in the overexpression of FOXO3a, which triggers nucleotide binding domain-like receptor protein 3 (NLRP3) inflammasome activation in SKM-1 cells and subsequent IL-1β release [51]. Furthermore, in a TPO-induced BM fibrosis model, IDH2 mutation has been shown to promote inflammation through increased expression of S100A8/A9 levels and NF-*κ*B hyperactivation [52]. In the JAK2V617F knock-in mouse model, concurrent SRSF2^P95H^ and JAK2V617F mutations reduce TGF-β levels but increase S100A8 and S100A9 expression compared with those in mice with JAK2V617F alone [53]. Additionally, in ASXL1-deficient JAK2V617F mice, levels of TNF, CCL2, and CCL5 are elevated compared to those with JAK2V617F alone [9]. These findings underscore the complex interplay between genetic mutations and the inflammatory process in PMF. However, the precise molecular mechanisms underlying these interactions require further investigation to fully appreciate their role in the disease and to identify potential therapeutic targets.

#### 3.1.5. The Role of Inflammasomes

Inflammasomes, high-molecular-weight cytosolic complexes in immune cells, are pivotal for detecting pathogens and inducing cell death in infected cells. They are also essential for activating the inflammatory caspase-1. Upon activation, caspase-1 processes IL-1β and IL-18 into their mature, bioactive forms, thereby contributing to sustained cytokine release. The nucleotide-binding domain and leucine-rich repeat containing receptor (NLR) family members, including NLRP1, NLRP3, NLRC4, and absent in melanoma 2 (AIM2), are key components in the formation of inflammasomes [54]. Furthermore, the activation of AIM2 or NLRP3 inflammasomes triggers pyroptotic cell death, as a consequence of CASP1-dependent proteolytic gasdermin D cleavage [55]. In MPN patients, inflammasome-related genes including NLRP3, NF-*κ*B1, CARD8, IL-1β, and IL-18 are highly expressed in BM cells, with increased expression correlating with the JAK2V617F mutation, white blood cell counts, and splenomegaly [56]. The microarray analysis conducted by Liew et al. [57] identified numerous genes involved in the activation of the inflammasome, such as AIM2, IL-1β, and CASP1, which were significantly upregulated in the cells where JAK2V617F was induced. Furthermore, Liew et al. [57] experimentally demonstrated that AIM2, a cytosolic sensor that detects double-stranded DNA, is a downstream target of JAK2V617F in D9 cell line. The latest research results show that the AIM2 inflammasome exacerbates atherosclerosis in clonal hematopoiesis from the JAK2V617F mutation [35]. Inflammasome activation triggers an inflammatory response that can lead to pathological inflammation and tissue damage, making them an exciting new class of potential drug targets. Small-molecule inhibitors of the NLRP3 inflammasome, currently in clinical trials, have shown proof of concept that inflammasomes are druggable, prompting drug development programs to focus on other key inflammasome molecules [58].

#### 3.1.6. The Role of Additional Soluble Mediators

In addition to the well-established role of inflammatory cytokines, additional soluble mediators in the generation and maintenance of inflammatory state in MPN have recently gained attention. Damage-associated molecular patterns (DAMPs), released by necrotic cells, function as alarmin-endogenous danger signals that are recognized by innate immune system cells. They promote the recruitment of leukocytes and activate TLRs, which in turn trigger monocytes and macrophages to increase cytokine production [59, 60]. Furthermore, these signals are crucial in modulating immune responses and are intimately involved in the regulation of cancer progression [61], highlighting their significance in cancer biology and their potential as targets for therapeutic approaches.

Circulating DAMPs, including high mobility group box 1 protein (HMGB1) and S100A8/A9, are upregulated in patients with PMF [62]. Gene expression and proteomic analyses of CD34^+^ cells and granulocytes from patients across all MPN subtypes have demonstrated an upregulation of the S100A8 and S100A9 subunits [63]. A recent study, utilizing RNA sequencing, followed by validation at both protein and mRNA levels, revealed that S100A8 was specifically enriched in MPN-model cells harboring the CALR type 1 mutation (52 bp deletion) compared to those with the CALR type 2 mutation (5 bp insertion) [64]. In triple-negative PMF, increased MYC expression and activation of S100A9 due to trisomy 8 drive a complex inflammatory network that involves numerous hematopoietic cell types in the BM microenvironment. Genetic deletion of S100A9or targeting the MYC-S100A9 pathway effectively ameliorates BM fibrosis, underscoring the MYC-S100A9 as a potential therapeutic target for PMF [65]. Observational studies have revealed a strong correlation between plasma levels of S100A8/A9 and total white blood cell counts in MF patients, with monocytes emerging as a notable source of these proteins [62]. Additionally, the strong link between plasma S100A8/A9 and neutrophil counts indicates that neutrophils may contribute to elevated systemic levels of these proteins. These insights collectively suggest that both the malignant clone and normal cellular components, particularly monocytes, significantly contribute to the production of S100A8/A9.

A preclinical study employing single-cell RNA sequencing has identified the specific upregulation of S100A8/A9 transcripts within the BM MSCs and megakaryocytes in a TPO-induced BM fibrosis model [20]. This finding suggests that, under the stress of BM fibrosis, wild-type MSCs act as a source of the alarmin complex. Tasquinimod, a small molecule inhibitor of S100A8/A9 signaling, has demonstrated its ability to alleviate myeloproliferation and BM fibrosis in retroviral models of JAK2V617F. This highlights S100A8/A9 as a promising therapeutic target for PMF. Additionally, a Phase 2 clinical trial is currently underway for patients with MF (NCT06327100).

In conclusion, multiple pro-inflammatory signaling pathways, including JAK-STAT, NF-*κ*B, and those associated with genetic mutations, along with the actions of inflammasomes and soluble mediators like DAMPs, intricately interact to drive the inflammatory state in PMF. Understanding these complex mechanisms is crucial for the development of more effective therapeutic strategies. Future research should focus on further elucidating the precise molecular mechanisms and identifying additional potential therapeutic targets within these pathways.

### 3.2. Cellular Sources of Inflammatory Mediators in PMF

Pro-inflammatory cytokines, produced by malignant HSPCs and their descendants, notably monocytes and megakaryocytes, interact with surrounding nonclonal hematopoietic and nonmutated stromal cells, thereby stimulating them to produce additional cytokines, leading to sustained high levels in the BM and peripheral blood (PB) [6]. Therefore, cellular drivers of inflammatory mediators encompass both malignant HSPCs and their descendants, as well as nonclonal hematopoietic cells [14]. As previously mentioned, MSCs in the fibrotic BM serve as a source of S100A8/A9 [20]. BM MSCs are known to exert both inflammatory and anti-inflammatory effects. Specifically, MSCs have been shown to suppress T cell proliferation and natural killer (NK) cell cytotoxicity through the expression of inflammatory cytokines, including IL-10, indoleamine 2,3-dioxygenase 1, and TGF-β [66]. Moreover, recent studies have underscored the pivotal role of TGF-β signaling in MSCs in the genesis of BM fibrosis [18]. Consequently, nonmutated and nonhematopoietic stromal cells, far from being mere recipients of pro-inflammatory and profibrotic signals actively engage in cytokine production, thereby intensifying the inflammatory environment [20]. Subsequently, we will focus on the role of malignant HSPCs and their descendants in cytokine secretion, starting with megakaryocytes.

#### 3.2.1. Megakaryocytes

Megakaryocyte proliferation and morphological atypia, associated with reduced GATA1 protein levels, are characteristic features of PMF [67]. JAK2V617F-mutated megakaryocytes contribute to the expansion of HSCs [26] and the process of hematopoietic aging [68] in a transgenic mouse model with megakaryocyte-specific expression of the human JAK2V617F gene under the control of PF4 and FF1 promoters. Megakaryocytes derived from JAK2V617F-induced pluripotent stem cells and those found in PMF mouse models display a pro-inflammatory and profibrotic phenotype, along with reduced ribosome biogenesis [69]. At single-cell RNA level, megakaryocytes in nonfibrotic/non-PMF conditions are enriched for processes related to megakaryocyte differentiation, whereas those in fibrotic/non-PMF MPN are enriched for pathways associated with inflammation and fibrosis [70]. This indicates that megakaryocytes play an active role in the pathogenesis of inflammation and BM fibrosis. Research in MPN mouse models has established that megakaryocytes are pivotal in inducing BM fibrosis through the overproduction of profibrotic cytokines and growth factors such as TGF-β, beta fibroblast growth factor, vascular endothelial growth factor, and PDGF [18]. Targeting megakaryocytes has been shown to ameliorate disease symptoms in these models. Single-cell RNA sequencing analysis has been pivotal in revealing the transcriptional networks associated with profibrotic and pro-inflammatory megakaryocyte phenotypes, potentially unearthing new therapeutic targets. A study employing multimodal single-cell data has uncovered that the cellular and molecular basis of megakaryocyte differentiation is characterized by an early stem cell bias toward megakaryopoiesis, accompanied by unique molecular signatures associated with the disease [71]. In this study, G6B was identified as a pivotal molecular target through single-cell multiomics, with the potential to curb aberrant megakaryocyte differentiation and to target mutant clone-derived HSPCs and megakaryocyte progenitors, offering a potential therapeutic avenue [71, 72]. The neutrophil chemoattractant IL (CXCL) 4, a chemokine synthesized by megakaryocytes, plays a central role in platelet physiology. Its absence leads to reduced TPO-induced TGF-β expression [73], highlighting its regulatory role in TGF-β production in PMF. Although inhibiting CXCL4 alone does not fully reverse BM fibrosis, it significantly impacts the MPN phenotype, including inflammation, megakaryocyte dysplasia, and JAK-STAT activation, underscoring its importance in the pathophysiology of the disease [74]. TCF3, a transcription factor downregulated in MF megakaryocytes, may reveal new therapeutic targets by studying its role in fibrosis evolution [75]. The p19/CDK4/CDK6 axis, when deregulated in JAK2V617F megakaryocytes, accelerates BM fibrosis development [76]. In PMF, CD34^+^ cells overproduce the v-maf avian musculoaponeurotic fibrosarcoma oncogene homolog, which drives the differentiation of HSPCs into monocyte/macrophage and megakaryocyte lineages, contributing to the pathogenesis of disease [77]. Furthermore, PIEZO1, a mechanosensor upregulated in PMF, may contribute to abnormal megakaryopoiesis, highlighting the importance of extracellular matrix sensors in megakaryocyte function and platelet production and suggesting potential for targeted therapies in PMF [78]. These molecular targets offer potential intervention points for abnormal megakaryocyte differentiation observed in PMF.

#### 3.2.2. Monocytes

Elevated monocyte counts in the PB have been recognized as a potent and independent predictor of poor survival in PMF, as reported by [79]. Multiple studies have converged on this finding, highlighting the significance of monocytes in the disease prognosis. CD14^+^ myeloid cells are the principal cellular source of the constitutively overproduced cytokines in PMF [41], particularly TNF [80]. Proper TLR signaling is regulated by negative feedback, with IL-10 being a key inhibitor. However, MPN patient monocytes show a functional defect in responding to IL-10, leading to aberrant TLR signaling and increased TNF production [80]. Single-cell RNA transcriptomes of BM from retroviral models of MPLW515L have revealed that monocytes/macrophages are significantly enriched in IL-4 and TNF, underscoring their role in the inflammatory processes of PMF [5].

PB monocytes can be immunophenotyped into three subsets: classical (CD14^+^ CD16^−^), nonclassical (CD14^−^CD16^+^), and intermediate (CD14^+^ CD16^+^). The intermediate monocyte subset, important in inflammation-related diseases, is significantly elevated in various MPN subtypes. The JAK2V617F mutation is strongly linked to the proportion of this intermediate subset [81]. High CD163 expression in macrophages is indicative of tissues undergoing an inflammatory response [82]. In patients with PMF, there is a notable increase in CD163^+^ monocytes/macrophages in the BM, which positively correlates with decreased hemoglobin levels. Further research is needed to investigate the role of CD163 in monocytes/macrophages in PMF, as it may represent a potential therapeutic target for anti-inflammation drugs.

In PMF, research on JAK2V617F transgenic mice shows neoplastic monocyte-derived fibrocytes in the BM, and spleens are major collagen and fibronectin producers, with TGF-β1 signaling promoting their differentiation. Depleting JAK2V617F and CD11b^+^ monocytes can remove fibrocytes, marking neoplastic monocytes as a potential PMF target [83].

Pentraxin-2 can prevent monocyte-to-fibrocyte differentiation [84], and its recombinant form zinpentraxin alfa shows preclinical antifibrotic activity [85]. Furthermore, monocytes with JAK2V617F express the signaling lymphocyte activation molecule F7 (SLAMF7), linked to BM fibrosis [86]. Anti - SLAMF7 antibodies, which can inhibit monocyte-derived fibrocyte differentiation, offer a promising treatment [86]. More recently, Bing Li et al. analyzed clinical data from 387 patients with PMF and explored the pathogenesis using an inducible NRAS^G12D^ and JAK2V617F knock-in mouse model [87]. Their results show CD38 is crucial for monocyte differentiation and fibrogenesis, and inhibiting CD38 or supplementing with nicotinamide mononucleotide can reduce fibrocyte formation, delay fibrosis, and extend survival, suggesting CD38 as a novel target for treating MF.

#### 3.2.3. Neutrophils

In PMF patients with the JAK2V617F mutation, BM granulocytes exhibit significant heterogeneity, marked by an over-maturation phenotype in granulocyte development [88]. Moreover, high expression of CD11b, a marker of neutrophil activation, and CD10 in granulocytes have been associated with a poorer prognosis [88], further emphasizing the significance of these cells in the disease course.

Recent studies utilizing the JAK2V617F knock-in model have confirmed that hematopoietic JAK2V617F expression leads to a high risk of cerebral venous sinus thrombosis, with neutrophils playing a crucial role, as evidenced by increased brain neutrophil infiltration and enhanced neutrophil activation in PB [89]. Further research utilizing mouse models has demonstrated that neutrophil-specific JAK2V617F expression, but not CALR^del^, is sufficient to induce the production of pro-inflammatory cytokines, including IL-1β [90]. Collectively, these findings strongly suggest that malignant neutrophils are key drivers in promoting inflammation within the context of MPN.

Upon stimulation, neutrophils initiate a cascade of intracellular signaling events involving reactive oxygen species (ROS), myeloperoxidase (MPO), neutrophil elastase, and protein arginine deiminase 4 (PAD4). These signals culminate in the release of extracellular chromatin decorated with histones and an array of granular proteins, effectively trapping and neutralizing microorganisms [91]. This process, known as NETosis, leads to neutrophil extracellular traps (NETs) formation. Notably, NETs can be triggered not only by microorganisms but also by a variety of stimuli, including cytokines like TNF-α and IL-8, activated platelets, and autoantibodies.

Plasma biomarkers indicative of NETosis, such as plasma free DNA and MPO–DNA levels, are significantly elevated in MPN patients. However, it is important to note that these elevations are not specific to a particular type of MPN or JAK2 allele burden [92]. For instance, hydroxyurea and IFN treatment can lower neutrophil counts without affecting elevated formation of NETs [92]. Additionally, PMF patients show increased basal ROS levels and NETosis in unstimulated neutrophils. Conversely, the induction of NETs by a potent inducer, phorbol myristate acetate (PMA), is impaired due to reduced PMA-triggered ROS production [93]. These collective findings suggest that the formation of NETs may be closely related to intrinsic changes within the malignant clone, rather than being mainly driven by other cytokines.

JAK2V617F knock-in mouse model exhibit heightened tendencies toward NETs formation and thrombosis, which can be reduced by the JAK2 inhibitor ruxolitinib [94]. This clearly links JAK2V617F expression to the formation of NETs. PAD4, vital for NETs formation, is upregulated in JAK2V617F-expressing neutrophils and is essential for NET formation associated with this mutation [94]. The insufficiency of mutated neutrophils alone to initiate NETosis is highlighted by research findings. For example, in PF4iCre; Jak2V617F mice, an enhanced NETosis response was observed, a phenomenon that was not present in MRP8Cre; JAK2V617F mice. This strongly indicates that additional factors are necessary for NETosis to occur. Ex vivo studies have further demonstrated that the interaction between JAK2V617F-mutated neutrophils and platelets is essential for the formation of NETs, emphasizing the collaborative role of platelets in this process [95].

Excessive NETs can cause tissue damage and play a role in the pathogenesis of metabolic, autoimmune, and autoinflammatory diseases, as well as certain septic conditions [96]. Utilizing mouse model experiments, Tripodo and colleagues demonstrated that NETs contribute to the progression of myeloid malignancies by inducing immune and inflammatory alterations within the BM microenvironment. Specifically, the formation of NETs via IL-6, stem cell factors, and NF-*κ*B signaling pathways can elicit the proliferation of HSCs [96]. Some studies have also established a link between NETs and fibrotic processes, suggesting that the interaction of NETs components and potential defects in NET clearance can impact fibroblast activation, driving the disease toward fibrosis [97]. It is well-recognized that NETs contribute to the prothrombotic state [94]. Importantly, studies have also demonstrated that inhibiting NET formation can reduce the risk of thrombosis [98]. This indicates that further research into the mechanisms of NETs formation and their role in MPN could potentially lead to the development of novel therapeutic strategies.

#### 3.2.4. Mast Cells

Mast cells play a crucial role in the development of both innate and adaptive immunity [99]. An initial study identified a significantly higher mast cell count in the fibrotic BM of PMF patients, with advanced fibrosis correlating with increased mast cell infiltration positive for TGF-β and IL-13 [100], suggesting their role in PMF pathogenesis. Subsequent research using single-cell RNA sequencing in MPLW515L and JAK2V617F mouse models confirmed a marked increase in mast cells, identified as the primary producers of IL-13, a cytokine that promotes mutated megakaryocytes growth and triggers TGF-β surface expression and collagen synthesis [101]. More intriguingly, a recent study employing single-cell transcriptomics analysis in the MPLW515L mouse model has highlighted a significant increase in basophils and mast cells, underscoring their critical interactions with megakaryocytes, MSCs, and pro-inflammatory fibroblasts in the formation of a TNF signaling hub [5]. These findings underscore the roles of mast cells in the pathogenesis of PMF.

## 4. Cellular and Molecular Mechanisms of Immune Dysregulation in PMF

### 4.1. Manifestations of Immune Dysregulation in PMF

Recent single-cell RNA sequencing analysis of BM aspirates from PMF patients has uncovered that immune cells in overt-PMF display unique signatures of immune dysfunction and suppression, contrasting with those in the pre-PMF phase [70]. These findings underscore the intricate interplay between malignant clones and nonmalignant immune cells within the BM microenvironment, further validating the notion that immune dysregulation plays a substantial role in disease progression [102]. Immune dysregulation in PMF patients is characterized by a range of abnormalities affecting both the innate and adaptive immunity. This includes the downregulation of human leukocyte antigen-I and human leukocyte antigen-II expression, which impairs antigen presentation and T cell activation [103]. Concurrently, the activation and differentiation of lymphocytes are compromised [32], leading to a diminished immune response to pathogens [104, 105]. Furthermore, there is a notable reduction in the frequency and effector functions of NK cells [106], along with a decreased count of effector T cells in the PB [107] and other T lymphocyte populations [108, 109]. This dysregulation impedes the recognition and elimination of malignant cells, weakening immune surveillance and facilitating clonal evolution [102]. Therefore, understanding and addressing the impact of immune dysfunction on PMF are crucial for developing effective therapeutic strategies that target not only the malignant clone but also the immune dysregulation that accompanies the disease.

### 4.2. Mechanicm of Immune Dysregulation in PMF

IL-6, a crucial cytokine in PMF, is produced aberrantly via the JAK-STAT pathway, influencing inflammation, immune response, and hematopoiesis. It plays a key role in homeostasis and disease resolution [110]. The JAK-STAT pathway negatively regulates the functions of neutrophil and NK cell [111], key for innate immunity, and suppresses effector T cell activities, vital for adaptive immunity. STAT3 also inhibits the maturation and function of dendritic cells, key for antigen presentation and T cell activation, while promoting the expansion of regulatory T cells (Treg) and myeloid-derived suppressor cell (MDSC) populations [112]. Mouse model experiments have implicated the IL-6/JAK/STAT3 signaling pathway in the induction of checkpoint inhibitory receptor PD-1 and/or its ligand, PD-L1 expression. Patients with PMF harboring the JAK2 mutation exhibit higher PD-L1 expression in megakaryocytes, monocytes, granulocytes, MDSCs, and platelets [113]. Cellular experiments have confirmed that in JAK2V617F-mutant cells, STAT3/5 phosphorylation, driven by oncogenic JAK2 activity, enhances PD-L1 promoter activity and PD-L1 protein expression, which inhibits T cell activity [36], downregulates antitumor immunity, and promotes tumor invasion. Studies have indeed highlighted a compromised T cell phenotype in MPN, with increased PD-1 expression on T cells [114, 115].

### 4.3. Therapeutic Exploration for CALR Mutations and T Cell Immunity

Special attention has been given to patients with CALR mutations, as those with frameshift mutations in the CALR gene exhibit a common 36-amino acid sequence at the C-terminus of the mut-CALR protein [116]. This sequence creates a neoepitope on MPN cells, enabling therapies to selectively target mutCALR-expressing cells. Such treatments could reduce the mutCALR allele burden and potentially eliminate the mutant clone without disrupting normal hematopoiesis, thereby improving disease outcomes. INCA033989 inhibits abnormal TPOR signaling in mutCALR-expressing MPN cells without affecting normal hematopoiesis [117], validating its therapeutic potential as a targeted therapy for MPN that does not interfere with normal hematopoiesis.

Regarding T cell immunity, some patients accumulate specific T cell responses against mut-CALR. However, T cells from CALR-mutant patients display increased expression of checkpoint inhibitory receptor cytotoxic T lymphocyte-associated antigen-4 (CTLA-4), which hinders the ability of the immune system to target and eliminate malignant clones expressing this mutation. In vitro blockade of PD-1 and CTLA-4 by monoclonal antibodies and in vivo PD-1 blockade by pembrolizumab can revive mut-CALR-specific T cell immunity [118], indicating a potential benefit from immune checkpoint inhibitors (ICIs). However, a Phase 2 trial of pembrolizumab in advanced MF patients did not yield objective clinical responses. A new trial (NCT05393674) is currently recruiting to explore the combination of nivolumab and fedratinib in MF patients.

An observational study [119] has employed flow cytometry to conduct an extensive analysis of lymphocytes in JAK inhibitor-naïve MF patients, irrespective of their mutational profiles, with the aim of identifying a broader range of checkpoint inhibitory receptors beyond the well-known PD-1 and CTLA-4. The study findings indicate that, in addition to PD-1 and CTLA-4, there is a significant upregulation of other immune checkpoint receptors on cytotoxic T cells. These receptors include lymphocyte activation gene-3, T cell immunoglobulin and mucin domain 3 (TIM-3), CD160, and CD244, which are associated with a reduced ability to produce TNF and IFN-γ. Furthermore, an increase in CTLA-4 ligands, CD80 and CD86, was observed on granulocytes and monocytes in MF, pointing to a potential role of myeloid cells in the suppression of T cell activity. The study also demonstrated that anti-CTLA-4 therapy could enhance the ability of cytotoxic T cells to eliminate neoplastic monocytes and granulocytes in coculture systems, suggesting that CTLA-4 blockade may augment immune responses against tumor cells. This finding warrants further clinical trials on CTLA-4 antibodies. The results of a trial involving sabatolimab, an anti-TIM-3 monoclonal antibody (NCT04097821), are eagerly awaited to provide additional insights into the potential of these therapies.

### 4.4. TGF-β Mediated Immune Dysregulation Triggered by Oncogenic Signaling Pathways

While we have delved into the role of immune checkpoint receptors in PMF, there is another crucial aspect of immune dysregulation that remains to be explored. One of the unanswered questions regarding PMF immune dysregulation is how malignant HSCPs contribute to the disruption of immune homeostasis. Single-cell RNA sequencing has played a crucial role in uncovering the molecular underpinnings by which oncogenic signaling facilitates immune evasion. TGF-β has been identified as a key factor in this process, promoting T cell exclusion and hindering immune response development within the tumor microenvironment [120]. Research has shown that the CALRdel52 mutation leads to an expansion of TGF-β1-producing erythroid progenitor cells and promotes the expansion of FoxP3^+^ Treg in a CALR knock-in mouse model [121]. Blocking TGF-β with an anti-TGF-β antibody has been demonstrated to improve survival in these mice and to enhance glycolytic activity in CD4^+^ and CD8^+^ T cells. Furthermore, the absence of T cells also negates the benefits of TGF-β neutralization, underscoring the pivotal role of T cells in this immune response. In vitro experiments have demonstrated that TGF-β1 reduces T cell production of perforin and TNF, further illustrating its suppressive effects on T cell function. The CALRdel52/ERK/Sp1 pathway, which is responsible for TGF-β1 production by CALRdel52 cells, has been identified as a potential therapeutic target for mitigating the immunosuppressive effects of TGF-β in CALRdel52 MPN.

### 4.5. The Role of MDSCs

MDSCs, which are pathologically activated neutrophils, form a heterogeneous population that expands in PB and lymphoid tissues during inflammatory conditions, such as infections and traumas. These cells are particularly noted in tumor-bearing mice for their T cell suppressive functions and are a critical component of the tumor microenvironment, playing crucial roles in tumor progression and therapy resistance [122]. Research shows that subjects with JAK2 mutations present an increased proportion of circulating MDSCs, which correlates with disease progression, substantial immunosuppressive mechanisms, and distinctive tumor-promoting and metastatic attributes [123]. MDSCs from patients with MPN have a greater inhibitory potential against autologous CD3^+^ T cells compared to MDSCs from healthy donors [124].

## 5. Imbalance Between ROS Production and Defensive Pathways in PMF

The role of ROS with consequent DNA damage is now recognized as a hallmark of cancer. Under physiological conditions, a delicate balance between pro-oxidant and antioxidant factors is essential for maintaining proper cellular functions. Disruption of this equilibrium leads to oxidative stress, which in turn triggers the pathological accumulation of ROS. ROS, considered as secondary byproducts of oxygen consumption and cellular metabolic processes, arise from the incomplete reduction of molecular oxygen, primarily produced by neutrophils, macrophages, and monocytes.

### 5.1. Sources and Regulatory Mechanisms of ROS Production in PMF

PMF patients are characterized by increased ROS production [125, 126], a phenomenon attributed to multiple factors. In the subsequent sections, we provide an in-depth analysis of the sources and regulatory mechanisms that govern ROS production in PMF.

Driver mutations in JAK2, as demonstrated in knock-in mouse models and CD34^+^ cells from MF patients, induce ROS accumulation [127]. Mice studies have shown that the antioxidant N-acetylcysteine can reduce splenomegaly, JAK2-mutant HSCPs, and normalize blood parameters [127]. This indicates a clear link between JAK2 mutations and enhanced ROS levels. CALR mutations not only induce ROS accumulation but also increase sensitivity to oxidative stress, resulting in increased oxidative DNA damage [128]. Here, the dual impact of CALR mutations on ROS production and cellular susceptibility to oxidative stress further emphasizes the significance of genetic factors in ROS-related pathogenesis in PMF

The activation of the NF-*κ*B pathway by oxidative stress perpetuates a cycle of further ROS production [129]. This self-perpetuating loop highlights the complex feedback mechanisms at play. Cytokines, particularly TGF-β, play a significant role in the generation of ROS within the malignant clone. TGF-β upregulates the expression of miR-382-5p and concurrently diminishes the activity of superoxide dismutase 2, leading to an increase in ROS levels [130]. Galunisertib, a TGF-β receptor type 1 inhibitor, has emerged as a potential antineoplastic agent capable of reducing oxidative stress. It can restore superoxide dismutase 2 activity and reduce ROS accumulation by targeting miRNA expression [130].

Lipocalin-2, a pro-inflammatory mediator that is increased in PMF patients, has been shown to further increase ROS levels, leading to DNA strand breaks and apoptosis in normal CD34^+^ cells, whileMF CD34^+^ cells appear to be resistant to these effects [131]. Understanding the differential response between normal and myelofibrotic CD34^+^ cells could offer insights into the unique biology of PMF cells. Additionally, the accumulation of iron in PMF patients, often resulting from frequent blood transfusions, can intensify ROS levels [132]. This external factor further contributes to the oxidative stress burden in PMF patients.

### 5.2. Multifaceted Roles of ROS in PMF Development

ROS are master regulators of cancer development that act through complex mechanisms. Initially, ROS can oxidize cellular components, including proteins, lipids, and double-stranded DNA, which results in heightened genomic instability and promotes cellular transformation, contributing to cancer initiation [133].

In addition, ROS initiate inflammatory pathways and protumorigenic pathways such as p38-mitogen-activated protein kinase, protein kinase B/mammalian target of rapamycin, and JAK/STAT, leading to aberrant cell proliferation, metastasis, resistance to apoptosis, angiogenesis, and other processes crucial for MPN onset and progression [134].

Hypoxia inducible factor-1α (HIF-1α), a master regulator of response to hypoxia and various genes involved in proliferation, survival, chemoresistance, and metabolism, is upregulated in JAK2V617F^+^ cells under normoxic conditions [135]. The ROS-mediated induction of HIF-1α is indicated by the decrease in HIF-1α protein levels upon treatment with the antioxidant Nrf2 activator dimethyl fumarate. Pharmacological inhibition and knockdown HIF-1α in JAK2V617F^+^ cells lead to impaired growth and survival, induced apoptosis, and cell cycle arrest, specifically in JAK2V617F^+^ cells, highlighting HIF-1α as a potential therapeutic target in JAK2V617F^+^ MPN [135].

Notably, that in a highly oxidative BM environment, the accumulation of ROS disrupts the balance between HPSCs and MSCs, causing MSCs to deteriorate and age, and initiating a self-reinforcing cycle that generates even more ROS [136] and primes HPSCs for transformation [137, 138]. This disruption of the stem cell niche has far-reaching implications for the overall hematopoietic function. Furthermore, ROS also impact T cells activation, differentiation, and death [139]. As T cells are crucial for immune surveillance, ROS-induced changes in T cells can affect the ability of the body to recognize and eliminate cancer cells.

**Figure 1:**
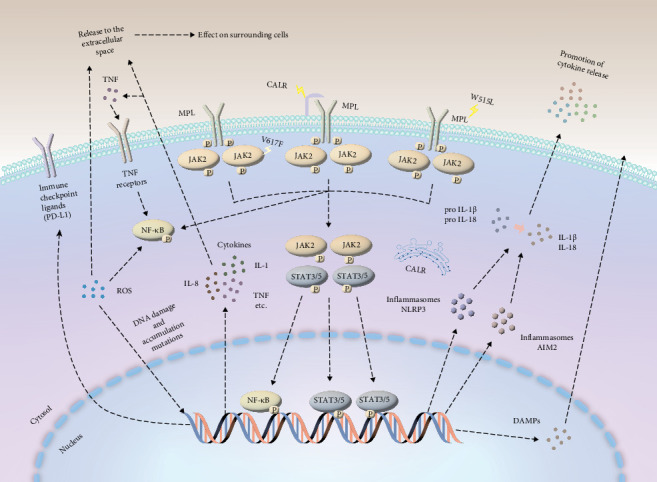
Molecular pathways propelling pro-inflammatory processes in neoplastic clones. The molecular mechanisms propelling pro-inflammatory processes within neoplastic clones in PMF are multifaceted. Driver genetic mutations in Janus kinase signal transducer JAK2 (JAK2V617F), the chaperone protein calreticulin (CALR), or the thrombopoietin receptor (MPL) disrupt the Janus kinase-signal transducer and activator of transcription pathway. This disruption leads to excessive production of inflammatory mediators, heightened oxidative stress, and immune system deregulation. NF-*κ*B signaling is hyperactivated by both cell-autonomous signaling from mutant JAK2 kinase and noncell autonomous activation by inflammatory mediators, with TNF playing a pivotal role in this inflammatory cascade. Inflammasomes are also involved, processing IL-1β and IL-18 into their mature, bioactive forms, which contributes to sustained cytokine release. Furthermore, damage-associated molecular pattern, particularly S100A8/9, has emerged as significant soluble mediators in the initiation and maintenance of the inflammatory state in PMF.

In summary, ROS are indeed pivotal in cancer incidence and progression, prompting significant interest in their potential as therapeutic targets. This has instigated substantial interest in exploring their potential as therapeutic targets. Antioxidants, by virtue of their capacity to scavenge free radicals and forestall oxidative stress, have emerged as prospective therapeutic agents. Nevertheless, further in-depth research is imperative to comprehensively elucidate the molecular mechanisms underpinning ROS production and antioxidant depletion.  Figure 1 encapsulates the intricate cellular and molecular pathways responsible for the overproduction of inflammatory mediators, elevated oxidative stress, and immune system dysregulation in PMF.

## 6. Anti-Inflammatory Strategies in PMF

### 6.1. JAK Inhibitors and Combination Therapy With New Agents in PMF Treatment

JAK inhibitors, with ruxolitinib at the forefront, assume a central role by targeting the deregulated JAK-STAT signaling pathway. Ruxolitinib was the first JAK inhibitor approved by the U.S. Food and Drug Administration (FDA) in 2011 for the treatment of intermediate- or high-risk MF and remains the standard first-line therapy due to its proven efficacy in reducing splenomegaly and disease-related symptoms. Newer-generation JAK inhibitors have also become accepted treatment options. In addition to ruxolitinib, several newer-generation JAK inhibitors have been approved by the U.S. FDA for the treatment of intermediate- to high-risk MF, each with specific indications and patient profiles. Fedratinib was approved in 2019 as a first-line or second-line option, particularly for patients who are resistant or intolerant to ruxolitinib. Pacritinib, approved in 2022, offers a unique treatment avenue for MF patients with severe thrombocytopenia, a population for whom other JAK inhibitors are often contraindicated. Most recently, momelotinib received FDA approval in 2023 for patients with symptomatic anemia, with demonstrated efficacy in improving both anemia and splenomegaly, making it suitable as either a first- or second-line therapy. These agents expand the therapeutic landscape of MF, enabling more personalized treatment strategies based on hematologic parameters and treatment history. A variety of novel and promising therapeutic agents targeting apoptosis, epigenetic modulation, and signal transduction are currently being explored in preclinical and clinical settings [140].  Table 1 summarizes the ongoing clinical trials in MF.

In the pursuit of enhancing treatment efficacy, combination therapies for PMF are being actively investigated to enhance treatment efficacy by targeting multiple pathways involved in the disease. For instance, targeting inflammatory signaling pathways, such as NF-*κ*B with BET inhibitors CPI-101, has been shown to significantly reduce inflammatory cytokines when combined with JAK2 inhibitors. This approach provides a comprehensive strategy for the management of PMF [49]. INCB057643, another BET inhibitor, has shown good tolerability in patients, both as monotherapy and in combination with ruxolitinib. No treatment-related fatal events have been observed. Patients receiving either monotherapy or combination therapy have experienced improvements in anemia, spleen size, and symptom burden [141].

### 6.2. Immunomodulatory Therapies and Their Potential in PMF

Immunomodulatory therapies and immune system-targeting agents hold great promise in addressing the established dysregulated immune state in PMF [142]. The preliminary analysis of a study evaluating CK0804, a CXCR4 enriched T regs cell therapy added to ruxolitinib, shows initial safety with no myelosuppressive adverse events and promising clinical activity [143]. The study is actively enrolling participants (NCT05423691). JNJ-88549968, a CALRmutxCD3 T cell redirecting antibody, selectively targets CALRmut, with the potential to cure MPN by eliminating cancer clones. This antibody works by bridging CALRmut MPN cancer cells and T cells, inducing T cell activation and cytotoxicity against CALRmut cells both in vitro and in vivo [144]. Currently, JNJ-88549968 is advancing to clinical investigation in MPN patients (NCT06150157).

Therapeutic cancer vaccination with a peptide derived from CALR exon 9 mutations, or the mutant-CALR peptide vaccine combined with keyhole limpet hemocyanin and poly-ICLC, can induce strong cellular immune responses in patients with CALR-mutant MPN. This helps the immune system destroy cancer cells and improve outcomes in CALR mutation+ MPNs. These vaccines are currently under clinical investigation in MPN patients (NCT05025488 and NCT05444530).

### 6.3. IFN and Anti-Inflammatory Therapy With Recombinant IFN-α (rIFN-α)

IFN constitutes a family of more than 13 cytokines that are generated in response to the presence of intracellular pathogens. During infection, the primary soluble factors produced are of the α- and γ-types. Both classes of IFNs elicit an antiproliferative, apoptotic-inducing antiviral state, effectively thwarting the ability of intracellular pathogens to command host cells [145]. In an earlier mouse experiment, IFN-α was found to increase HSPCs proliferation through the IFN-α/STAT1 pathway [146]. Given that the expansion of HSPCs results in their widespread dysfunction [147], the stimulation of the IFN-α signaling cascade, in turn, leads to a decreased abundance and impaired functionality of HSPCs [146]. IFN-γ has also been demonstrated to modulate the proliferative capacity and migratory behavior of HSPCs [148].

IFN-α therapy is pivotal in part due to its anti-inflammatory ability to inhibit IL-1β and the production of cytokines it induces, as well as to block the NF-*κ*B signaling pathway. This mechanism underlies the potential of IFN-α to achieve long-term complete remission in MPN with JAK2V617F and CALR mutations [149, 150]. In experimental therapies, recombinant rIFN-α has demonstrated the ability to normalize BM structure by modulating immunity, and a clinical benefit has been observed in a majority of patients diagnosed with early-stage PMF [151, 152]. Moreover, combination therapy with IFN and JAK2 inhibitors has been demonstrated to elicit a regression of BM fibrosis in patients with MPN [153, 154]. One study revealed that individuals undergoing therapy with rIFN-α2 exhibit a gut microbiota composition that more closely resembles that observed in healthy subjects [155]. Interestingly, IFN-α2 treatment supports the preservation of a healthier microbiota, thereby suppressing PMF-induced microbial inflammatory signals [156]. There is a proposal advocating for a shift in the therapeutic paradigm, transitioning away from the conventional “monitor-and-observe strategy” toward an “initiative of prompt intervention,” where IFN-α2 serves as a fundamental medication for initial therapeutic intervention, beginning at the point of diagnosis [24].

### 6.4. Targeting Cytokines and Chemokines in PMF

Cytokines and chemokines serve as pivotal effectors in the intricate crosstalk between malignant HSPCs and BM stromal cells within the context of PMF. This interaction clearly highlights the potential of anticytokine and antichemokine therapy as a treatment modality for PMF.

Drawing insights from other disease models, anticytokine therapies have established efficacy in managing autoimmune diseases [157]. These autoimmune conditions are typified by intricate cellular and cytokine-mediated processes. In such diseases, targeting a single cytokine can trigger a “cascade” effect. This effect disrupts the entire sequence of inflammatory responses, leading to disease modification. The underlying principle lies in the fact that cytokines do not act in isolation; substantial evidence indicates that they exhibit synergistic effects rather than simply additive ones.

When extrapolating this concept to cancer, and specifically PMF, an imbalanced cytokine profile has been well-established as a contributing factor to cancer initiation and progression. In PMF, cytokines can promote chronic inflammation, which provides a fertile ground of malignant cell growth. Additionally, they can facilitate immune evasion, allowing cancer cells to escape the immune surveillance for the body. Given these roles of cytokines in PMF pathogenesis, anticytokine therapies present a promising approach for treating this disease [157].

Based on the promising potential of anticytokine therapies in PMF, researchers have been actively exploring relevant treatment agents. Shifting focus to current research efforts, several therapeutic agents are at the forefront of investigation. For example, luspatercept [158, 159] and KER-050 [160] specifically target the TGF-β pathway, while reparixin [161, 162] targets the CXCR1/2 pathway. Both KER-050 and reparixin are currently under clinical investigation in patients with MPNs (NCT05037760 and NCT05835466). The intricate cytokine-receptor network in PMF, featuring pivotal cytokines like TNF, IL-1β, TGF-β, and IL-8, highlights the need for precision medicine that accounts for individual patient features and tumor profiles. The identification of predictive biomarkers to select patients for cytokine-targeted therapies is essential for personalizing cancer treatment.

In anticytokine therapy, neutralizing antibodies and soluble receptors (IL receptor antagonists) have shown efficacy in blocking cytokine activity. Additionally, kinases downstream of cytokine receptors are being targeted to manage inflammation. Our subsequent analysis delves into the key mediators in PMF progression, examining their mechanisms and the potential for anticytokine therapies.

#### 6.4.1. Cytokine and Signaling Pathways Involved in the Origins of Myofibroblasts

The results of experiments conducted by Puneet in mice have clearly demonstrated the significant role of CXCL12-expressing MSCs in controlling BM ecological niche [163]. Complementary to this, Aoki demonstrated through his research that malignant clonal hematopoiesis leads to reduced expression of CXCL12 [164]. There is also evidence suggesting that the expression of CXCR4, the major receptor for CXCL12, is reduced on HSPCs in patients with MPN, which further reduce CXCL12 signaling [165]. Cellular experiments conducted by Lorena, in the context of JAK2 mutation, showed that the targeted deletion of CXCL12 in nestin+ cells augmented the growth of JAK2-mutated clonal populations [34]. Collectively, these findings suggest that hematopoietic cells with JAK2 mutations can remodel the BM niche by diminishing CXCL12 expression levels and reducing the number of MSCs, thereby creating a BM microenvironment with decreased CXCL12 levels that support the survival and proliferation of malignant cells.

To further explore the origin of fibroblasts, genetic fate-mapping studies have been carried out and have identified LeptinR+ MSCs [166] in the TPO overexpression model of MPN as targets of inflammatory signaling that transdifferentiate into myofibroblasts. Similarly, by using the TPO overexpression model of MPN and the retroviral expression model of the JAK2V617F mutation, Gli1+ MSCs [167] were pinpointed as another source of myofibroblasts and a potential therapeutic target. This process entails a substantial transcriptional reprogramming, wherein these MSCs reduce their secretion of factors that support the hematopoietic niche and simultaneously upregulate the expression of genes associated with fibrosis and osteogenesis.

Furthermore, pharmacologic or genetic modulation of PDGFRα signaling in LeptinR^+^ cells has been shown to curb their expansion and mitigate MF [166]. This indicates that PDGFα signaling in LeptinR^+^ MSCs is essential for the pathogenesis of MF. Additionally, studies have confirmed that genetic elimination of Gli1^+^ MSCs or pharmacological inhibition of the hedgehog–Gli signaling pathway with GANT61 ameliorates fibrosis in mouse models of MF [167]. CXCL4 signaling in Gli1^+^ MSCs has also been implicated in the development of MF [73, 167].

The identification of LeptinR^+^ and Gli1^+^ cells as potential MSCs that differentiate into myofibroblasts in PMF poses a crucial query within the domain: ascertaining the relative contributions of LeptinR+ MSCs, Gli1+ MSCs, and potentially other as yet undiscovered stromal cell populations to the progression of PMF. Researchers use single-cell RNA sequencing to gain a deeper understanding of the cellular mechanisms underlying fibroblast origin, as it provides in-depth insights into fate switching within the BM stromal compartment during PMF injury caused by driver mutations.

Leimkuhler et al. [20] conducted single-cell RNA sequencing analysis on the BM niche and identified two distinct MSC populations as the primary drivers of BM fibrosis in the TPO overexpression model and the retroviral expression model of the JAK2V617F mutation. Under steady-state conditions, these MSC populations, characterized by LeptinR+ with adipogenic or osteogenic bias, exhibit high expression of CXCL12, suggesting their role in supporting hematopoiesis. At the single-cell level, Gli1^+^ cells have been identified as MSCs that are pivotal in driving fibrosis, acting as fibrosis-driving cells [20].

However, Lei Ding et al., through single-cell RNA sequencing analysis of BM stroma from the TPO overexpression model and the retroviral expression model of the MPLW515L mutation, presented a somewhat different perspective. They found that virtually all myofibroblasts originated from LeptinR+cells [168]. These cells showed reduced expression of hematopoietic niche factors and increased expression of fibrogenic factors. Gli1-lineage cells, a minority among stromal cells, underwent myofibroblastic differentiation. Contrary to previous research [167], evidence from a study by Lei Ding demonstrated that the deletion of Gli1 from either the hematopoietic or stromal compartment did not impact PMF pathogenesis, including BM fibrosis. This discrepancy highlights an area of contention within the field. This suggests that while Gli1+ MSCs may contribute to the myofibroblast population, their role as the primary driver of BM fibrosis in PMF is still open to debate and requires further investigation. The study also revealed a significant expansion of pericytes and Sox10+ glial cells, and chemical or genetic ablation of BM glial cells ameliorated fibrosis in PMF models, indicating that Sox10+ glial cells are critical for PMF development [168].

A remarkable multiomics single-cell study, focusing on the retroviral expression model of the MPLW515L mutation, provided a comprehensive view of the intercellular communication within the BM in the context of MF. Within the stromal compartment, a significant expansion of LeptinR+ MSCs was observed, alongside a slight increase in fibroblasts and a relative decrease in chondrocytes, osteoclasts, and endothelial cells. Notably, this study diverged from earlier JAK2V617F knock-in models [83] as it failed to identify monocyte-derived fibrocytes within the myelofibrotic BM; no SLAM7 or monocyte-associated markers were detected among stromal cell subsets. Additionally, there was a notable increase in basophil and mast cell counts in MF mice. Aligning with prior studies [166, 167], MSCs in MF were found to prioritize the production of extracellular matrix components at the expense of hematopoietic support factors. Notably, this reduction was counterbalanced by an increased production of hematopoietic cytokines from basophils, mast cells, and specific pro-inflammatory BM fibroblasts [5]. A significant elevation in the expression of Lgals1, encoding the β-galactoside-binding protein galectin-1, was identified in MSCs, basophils, mast cells, and megakaryocytes. Inhibition of galectin-1 led to a decrease in myeloproliferation and fibrosis across in vitro and in vivo models and was associated with enhanced survival rates, underscoring its therapeutic potential.

In addition to the abovementioned cell types, research has also explored the role of other cells in fibroblast origin. Patients with PMF exhibit functional and morphological changes in microvascular endothelial cells within the BM and spleen, which are associated with a mesenchymal phenotype. This study is the first to propose that endothelial cells are a source of fibrous tissue in the BM [169]. Specifically, the loss of LATS2 kinase in endothelial cells initiates an endothelial-to-mesenchymal transition, which increases the expression of extracellular matrix components and secreted signaling molecules. The endothelial cell-specific inactivation of the Lats2 gene in Cdh5-CreERT2 transgenic mice results in osteosclerotic defects, BM fibrosis, extramedullary hematopoiesis, and splenomegaly, thereby identifying the endothelium as a central driver of BM fibrosis [170]. Moreover, increased numbers of macrophages, which can induce myofibroblast proliferation via vitamin D receptor signaling, have also been found in BM biopsies of PMF patients [171].

In summary, preclinical single-cell analyses have delivered high-resolution insights into PMF, elucidating the intricate cellular dynamics during myelofibrotic transformation and revealing numerous potential druggable targets. These studies have highlighted the involvement of MSCs, with a particular focus on the LeptinR^+^ subset, endothelial cells, macrophages, and Sox10^+^ glial cells. However, the field is faced with several challenges. The LeptinR^+^ cells are heterogeneous, comprising skeletal stem cells and progenitors of both osteogenic and adipogenic lineages [172]. Despite this heterogeneity, there is a scarcity of markers capable of distinguishing these subsets or comparing their functions. This lack of markers presents a significant challenge in unravlling the complex MSC population and the molecular pathways that govern their differentiation.

Furthermore, it is crucial to note that insights from single-cell analyses are often derived from retroviral models that lead to significant overexpression of JAK2V617F or MPLW515L, rather than from genetically engineered mouse models that more accurately mimic physiological levels of expression. Therefore, it is essential that key molecular targets identified in these models undergo validation in independent research teams and larger patient cohorts moving forward. Additionally, obtaining liquid BM in advanced MF is frequently difficult, which complicates the purification process necessary for single-cell RNA sequencing. In light of these caveats, future research in this field should address challenges by concentrating on developing more precise markers for LeptinR^+^ cell subsets, performing single-cell analyses in more physiologically relevant models, and conducting large-scale, multicenter studies employing serial primary samples to validate identified molecular targets. Moreover, the application of spatial transcriptomics to trephine biopsies from patients with PMF represents a cutting-edge approach that could greatly advance the exploration of the cytoarchitecture and molecular determinants underlying BM microenvironmental dysregulation in PMF.

#### 6.4.2. The Role of TNF and the Potential of Anti-TNF Therapy

TNF serves as a crucial instigator of hematological malignancies and has been implicated as a precursor to the BM failure syndrome Fanconi anemia [173]. The impact of TNF on HSPCs remains contentious. The response of HSPCs to TNF signaling can vary depending on the concentration of TNF, the duration of exposure, and the microenvironment where HSPCs are located. Furthermore, age-related differences in the responses of HSPCs to TNF may also exist [174].

As previously discussed, PMF is characterized by increased TNF levels. The Phase 3 COMFORT-I/II trials have demonstrated that inhibiting JAK1/JAK2 with ruxolitinib can significantly reduce TNF levels in patients with PMF. In a high-TNF environment, the physiological regulation of hematopoiesis by TNF is disrupted, conferring a selective growth advantage to JAK2V617F mutant over wild-type cells in vitro, thereby enabling clonal expansion [3]. Conversely, the absence of TNF attenuates disease phenotypes in JAK2V617F mice by restricting the expansion of clones [3, 175].

Furthermore, TNF signaling is intricately linked to the MF phenotype. TNF is known to exacerbate fibrosis by inducing the differentiation of monocytes into fibrocytes in both malignant and nonmalignant cells in ASXL1-deficient JAK2V617F mice [9]. The combination of the JAK inhibitor ruxolitinib and the TNF receptor antagonist R-7050 has been shown to reduce fibrocyte differentiation in vitro, highlighting its potential in managing the fibrotic processes associated with MF [9].

Over the past decade, TNF inhibitors have been successfully developed and applied in the clinical treatment of immunoinflammatory disease such as rheumatoid arthritis, psoriasis, Crohn disease, and ankylosing spondyloarthritis [176]. Clinical trials have also explored the use of TNF antagonists, such as the monoclonal antibodies infliximab and etanercept, in treating certain forms of cancer, including kidney and colorectal [176]. Notably, etanercept, a TNF neutralizing agent, has been shown to alleviate symptoms in myelofibbrosis patients [177]. However, as previously emphasized, the use of TNF scavengers, including etanercept and infliximab, increases the risk of opportunistic infections in MF patients due to their preexisting immune dysregulation.

Anti-TNF therapeutic strategies are increasingly focusing on the TNF receptor (TNFR), acknowledging the distinct roles of TNFR1 and TNFR2 in the pathobiology of JAK2V617-induced diseases [178]. In MPN mouse model, mouse progenitor cells with JAK2V617F downregulate XIAP and MAPK8 through a TNF/TNFR2-dependent autocrine loop, thereby escaping apoptotic responses and enhancing NF-*κ*B signaling [175]. This suggests that targeting TNFR2 could be a promising approach in treating PMF, as it may disrupt this loop and modulate the progression of the disease. Anti-TNFR2 treatment has been shown to selectively suppress myeloid colony formation of CD34^+^ cells isolated from patients with MF and murine Lin-Kit+ BM cells [89]. In a JAK2V617F knock-in mouse model, α TNFR2 antibody treatment decreased white blood cells and modulated the serum levels of CXCL2, CXCL5, IL-12, and macrophage colony-stimulating factor, indicating the potential efficacy of targeting TNFR2 in modulating immune and inflammatory aspects of the disease [178]. Treatment with α TNFR1 antibody treatment resulted in mild suppression of elevated hematocrit and attenuated splenomegaly. These findings underscore the importance of TNFR as a potential therapeutic target in the treatment of PMF, with a particular emphasis on TNFR2 due to its significant role in the pathogenesis of PMF and its potential as a therapeutic target.

#### 6.4.3. The Role of IL-1 and the Potential of Anti- IL-1 Therapy

Cellular experiments conducted by Pietras showed that IL-1 specifically targets HSPCs, enhancing their proliferation and stimulating the division and differentiation of these cells via the activation of PU.1-dependent molecular pathways [179]. In-depth investigations have revealed that the induction of PU.1 requires direct and prolonged signaling by IL-1, which is primarily facilitated through the activation of the NF-*κ*B pathway downstream of IL-1R, at the expense of other myeloid cell lineage expansions [179].

Wong et al. [11] utilized the NanoString technique to compare cytokine gene expression profiles in myeloid cells and found that the RNA transcript levels of pro-inflammatory cytokines especially IL-1β were elevated 1.5-fold in patients with PMF compared to those in pre-PMF patients, suggesting that IL-1β gene expression is associated with MF. Simultaneously, two separate studies reported the functional significance of the activation of the IL-1/IL-1R1 signaling pathway in MPN, with a particular emphasis on PMF. Patients with PMF exhibit increased expression of receptors related to IL-1β, along with an upregulation of target genes within the IL-1R signaling pathway [180].

The overproduction of IL-1β by the JAK2V617F mutant clone leads to neuropathy damage and compromises the survival of nestin+ MSCs, thereby affecting normal hematopoietic regulation [34]. In fact, IL-1β knock out in the hematopoietic cells of JAK2V617F mice not only reduces inflammatory cytokines but also prevents damage to nestin+ niche cells, which in turn reduces megakaryopoiesis. Consequently, this results in a decrease in MF and osteosclerosis. Moreover, the inhibition of IL-1β in JAK2V617F mutant mice by the anti-IL-1β monoclonal antibody 01BSUR also reduces MF and osteosclerosis [180]. Interestingly, when combined with ruxolitinib, an additive effect is observed [180]. These collective findings indicate that IL-1β plays a crucial role in promoting disease progression.

JAK2-mutant hematopoietic cells contribute to IL-1β production, thereby inducing an inflammatory environment that facilitates the expansion of JAK2V617F clones and the induction of MPN disease [27, 34]. However, IL-1β levels are not diminished by JAK1/JAK2 inhibition in MPN patients but suppressed by IRAK inhibition [181]. Notably, pacritinib, a JAK2 inhibitor that also targets IRAK1 [182], has been linked to a reduction in splenomegaly and alleviation of associated symptoms. It has demonstrated favorable tolerability, including mild myelosuppression, in two randomized Phase III trials [183]. Furthermore, CA-4948, another IRAK4 inhibitor, has shown potential in reducing leukemic engraftment in MF patient-derived xenograft mouse models [48]. Similarly, PF-06650833, an IRAK4 inhibitor, reduces inflammation in rheumatoid arthritis [184]. These results suggest that IRAK inhibitors may play a crucial role in managing inflammation and related symptoms in MF. However, their efficacy awaits further verification through clinical trials.

In this context, canakinumab, a fully human monoclonal antibody, targets the IL-1β pathway by blocking IL-1β interaction with IL-1R1, thereby inhibiting downstream inflammation and mediator production. It is currently under investigation in lower-risk myelodysplastic syndromes patients (NCT04239157) and has demonstrated efficacy in improving erythropoiesis and reducing transfusion dependence [185]. Additionally, the blockade of IL-1 using recombinant human IL-1 receptor antagonists, such as Anakinra, has been approved by the FDA for the treatment of rheumatoid arthritis, cryopyrin-associated periodic syndromes, and recurrent pericarditis. Rilonacept, another similar antagonist, has also been approved for the treatment of cryopyrin-associated periodic syndromes and recurrent pericarditis. These agents have also been considered for managing refractory cytokine release syndrome and immune effector cell-associated neurotoxicity syndrome, which are acute toxicities associated with chimeric antigen receptor T cell therapy [186]. The NLRP3 inflammasome, which is crucial for caspase 1 activation and thus IL-1β production, represents a promising therapeutic target for anti-IL-1β signaling strategies [58].

#### 6.4.4. The Role of TGF-β and the Potential Role of Anti-TGF-β Therapy

TGF-β is a pleiotropic cytokine known for its immunosuppressive, anti-inflammatory, and profibrotic activities. It comprises three distinct gene products: TGF-β1, -β2, and -β3. All are initially expressed in inactive (latent) forms, denoted as L-TGF-β, and activation is essential for their function. The transmembrane protein glycoprotein A repetitions predominant protein (GARP) binds and activates latent TGF-β1 on the surface of Treg cells and megakaryocytes or platelets [187]. Once activated, TGF-β interacts with the TGF-β receptor (TGF-βR) complex to regulate biological responses through drosophila mothers against decapentaplegic (SMAD) and non-SMAD pathways, which include the activation of extracellular signal-regulated kinase, c-Jun-terminal kinase (JNK), p38, PI3 kinase, and the NF-*κ*B pathway [188].

Elevated levels of TGF-β, produced by many kinds of hematopoietic cells, including fibrocytes [83], megakaryocytes, platelets, monocytes, immature myeloid cells, and B cells [41], are commonly observed in most patients with PMF [189]. Inhibition of TGF-β signaling, either genetically or pharmacologically, reduces reticulin fibrosis in the TPO overexpression and GATA1^low^ [190] models. The TGF-β receptor I kinase (ALK5)/Smad3 pathway is critical in the profibrotic activities mediated by TGF-β1 [189] Galunisertib, an ALK5 inhibitor, can partially alleviate MF in the retroviral expression model of the MPLW515L mutation and JAK2V617F transgenic mouse models [191]. However, studies have shown that pharmacological inhibition of TGF-β signaling alone is insufficient to completely reverse BM fibrosis. TGF-β signaling constitutes just one aspect of the multifaceted pathogenesis in BM fibrosis. Additional pathways, such as PDGFα signaling in LeptinR+ MSCs [166] and CXCL4 signaling in Gli1+ MSCs [167], also significantly contribute to the progression of BM fibrosis.

A recent study has highlighted the essential role of TGF-β signaling in MSCs in the development of BM fibrosis induced by MPLW515L and JAK2V617F [18]. Furthermore, noncanonical JNK signaling in MSCs has been identified as a mediator of the MF phenotype in the MPLW515L murine model. Treatment with SP600125, a JNK inhibitor, has been demonstrated to prevent MF in these mice. These findings underscore the potential therapeutic value of JNK inhibition in BM fibrosis progression in MPN. Interestingly, inhibition of TGF-β signaling in MSCs can block BM fibrosis. However, while blocking TGF-β signaling in MSCs halts BM fibrosis, it does not reverse the defective hematopoietic niche induced by MPLW515L, suggesting that distinct molecular mechanisms control fibrosis and niche cell fate.

In addition to its profibrotic role in PMF, TGF-β also exerts immunosuppressive functions in the pathogenesis of PMF. In vitro studies have shown that TGF-β reduces the cytotoxicity of T cells by decreasing the release of perforin and TNF [36]. In vivo targeting of TGF-β in a CALR knock-in mouse model has been found to enhance the glycolytic activity and TNF production of CD4^+^ T cells.

A preclinical study revealed that AVID 200, a capture agent for TGF-β1/β3 proteins, exerts beneficial effects on hematopoiesis and myelofibrotic conditions in patients with PMF [192]. Luspastercept, a recombinant fusion protein that interferes with TGF-β signaling, has been successful in reducing transfusion dependency in patients with transfusion-dependent myelodysplastic syndrome with ring sideroblasts [159] and thalassemia [158]. Ker-050 is a novel inhibitor of TGF-β family signaling, targeting multiple stages of the erythropoietic cascade [160]. This positions it as a promising therapeutic candidate for PMF, and it is indeed being evaluated in a Phase 2 clinical trial (NCT05037760).

Recent advancements in understanding TGF-β latency and activation have paved the way for improved therapeutic strategies targeting these pathways. In a mouse model of MPLW508A-induced PMF, increased GARP levels in the BM and spleen have been observed, primarily due to an increase in GARP + megakaryocytes. GARP is also expressed on Tregs. Using blocking anti-GARP:TGF-β1 monoclonal antibodies has been shown to reduce fibrosis and tumor burden by targeting the proliferation of MPLW508A-transformed cells. Importantly, the absence of GARP:TGF-β1 complexes on Tregs, compared to megakaryocytes, leads to reduced fibrosis and tumor burden, as seen in PMF mouse models with Treg- or megakaryocyte/platelet-specific GARP gene deletions. This suggests that TGF-β1 activation by GARP on Tregs plays a more significant role in PMF than megakaryocyte/platelet-derived TGF-β1. Anti-GARP:TGF-β1 monoclonal antibodies offer a potentially less toxic treatment alternative by selectively inhibiting TGF-β1 production from GARP-expressing cells, overcoming the toxicity issues associated with anti-TGF-β drugs [193].

#### 6.4.5. The Role of IL-8/CXCR1/2 Pathway and the Potential Role of IL-8/CXCR1/2 Therapy

The neutrophil CXCL8, which belongs to the CXC family of chemokines, is a versatile chemokine with potent pro-inflammatory properties. It attracts neutrophils to sites of inflammation, infection, or tissue damage, ultimately eliciting an acute inflammatory response. Recently, IL-8 has emerged as a pivotal player in tumorigenesis [194]. Its receptors, CXCR1 and CXCR2, are involved in the signaling pathways that recruit immunosuppressive MDSCs and autocrine growth of disease-initiating leukemic stem cells [195]. SX-682, an orally administered dual allosteric inhibitor of the CXCR1/2 receptors, has shown promising efficacy in treating patients with myelodysplastic syndromes who have not responded to hypomethylating agents [196].

In the context of MF, elevated serum CXCL8 levels have been linked to an increased risk of leukemic transformation and reduced overall survival in MF [6]. Dunbar et al. [197], using integrated single-cell transcriptional and cytokine assays, identified a strong enrichment of the CXCL8–CXCR2 pathway signature in MF, suggesting that this pathway promotes fibrotic progression. Various cell types within the PMF microenvironment produce and secrete IL-8 in an enhanced and deregulated manner, often mediated by CXCR2 and influenced by TNF/NF-*κ*B-regulated gene expression patterns [197].

Genetic deletion of CXCR2 in HSPCs of BM from retroviral models of MPLW515L resulted in reduced MF, prolonged overall survival, and a decrease in inflammatory signaling through the TLR pathway [197]. These genetic deletion findings were further supported by the pharmacologic inhibition of the CXCR1/2 pathway with reparixin. When combined with ruxolitinib, it shows potential in improving MF. In the Gata1^low^ mouse model of MF, studies have shown that CXCR1/2 inhibition using reparixin alleviates fibrosis by reducing TGF-β expression [161]. Specifically, malignant megakaryocytes in the mice model produce IL-8, which attracts neutrophils and initiates emperipolesis between them. This interaction increases the microenvironmental bioavailability of TGF-β, contributing to fibrosis. However, inhibition of CXCR1/2 by reparixin effectively reduces this TGF-β availability, thereby mitigating fibrosis [162]. These findings collectively validate CXCR1/2 as a promising therapeutic target for treating MF. Reparixin has entered clinical development for this condition, with its safety and efficacy currently being evaluated in patients who have undergone prior treatment, as well as in those who are ineligible or refuse treatment with JAK inhibitors (NCT05835466).

#### 6.4.6. The Role of IL-4/IL-13 Pathways in PMF

Among the abnormally expressed cytokines in PMF, the IL-4/IL-13 signaling axis has received attention due to its critical role in regulating the expression of TGF-β in models of pulmonary, cutaneous, and hepatic fibrosis, indicating its critical role in the fibrotic process [100, 198]. A study of BM biopsy specimens obtained from individuals diagnosed with PMF revealed an increase in the number of cells secreting IL-13 and TGF-β1, particularly mast cells, which constitute the primary source of IL-13 production [101]. Dupilumab is a therapeutic antibody targeting the IL-4 and IL-13 receptor subunit alpha used for the treatment of moderate–to–severe atopic dermatitis in adults and children [199].

### 6.5. Repurposing Existing Drugs and Exploring Plant Extracts

In addition to the abovementioned strategies, repurposing existing drugs with anti-inflammatory and antioxidant properties, such as statins [200] and histone deacetylase inhibitors [201], represents another therapeutic avenue.

**Figure 2:**
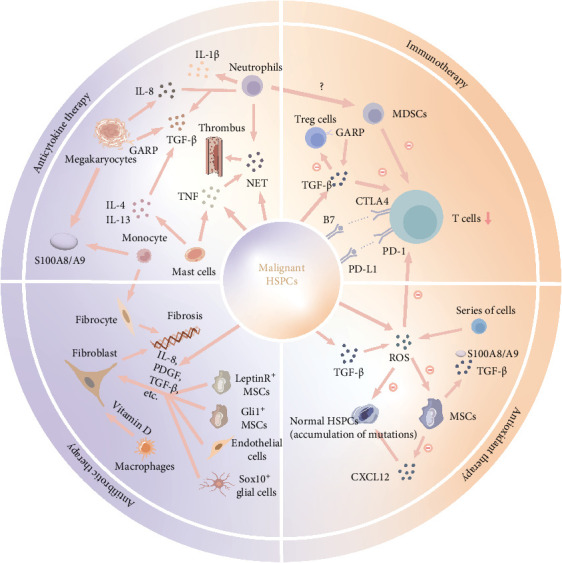
Graphic overview of the cellular and molecular targets of anti-inflammatory therapy in myelofibrosis. Emerging evidence has shown that the inflammatory state in myelofibrosis arises from three primary, intricately interconnected factors: the overproduction of inflammatory mediators, increased oxidative stress, and immune system dysregulation. Notably, cytokines play a pivotal role in the communication between the malignant clone and the bone marrow microenvironment, driving the central pathological change of myelofibrosis–bone marrow fibrosis. This figure provides a comprehensive overview and in-depth analysis of the cellular and molecular targets associated with inflammatory state. It establishes the rationale for anti-inflammatory therapy in myelofibrosis and encompasses a variety of strategies, including anticytokine, antifibrosis, antioxidative, and immunotherapy approaches.

Furthermore, various plant extracts and their bioactive components are also being explored for their potential anti-inflammatory, anti-antioxidant, and immunomodulatory effects in the treatment of cancer, including PMF [202].  Figure 2 offers an overview of the cellular and molecular targets in PMF therapy, encompassing a wide range of strategies including anticytokine, antifibrosis, antioxidative, and immunotherapy approaches.

### 6.6. Nonpharmacological Therapies

Engaging physical activity to mitigate cancer-related fatigue represents a burgeoning area of research [24] Yoga has demonstrated effectiveness in addressing stress and inflammatory responses associated with various cancers, such as breast, lung, and pancreatic, by enhancing the quality of life through alleviating stress, anxiety, depressive symptoms, and exhaustion, as well as improving emotional well-being and social interactions [203, 204]. A randomized clinical trial involving breast cancer patients revealed a trend toward decreased inflammatory cytokine concentrations in participants following yoga practice [205]. Physically active MPN patients also report less fatigue than their inactive patients do [206]. In two trials of MPN patients, nearly all reported that yoga practice had a beneficial effect on their well-being [205, 207]. A larger investigation on the corresponding effects in patients with MPN is forthcoming [208].

The Mediterranean dietary pattern has notable advantages in mitigating conditions pivotal to chronic subclinical inflammatory processes [207]. The anti-inflammatory characteristics of the Mediterranean diet are due to its high content of phenolic compounds and nutrient-dense nature [208]. The effectiveness of the Mediterranean dietary pattern in decreasing the levels of C-reactive protein and IL-6, consequently mitigating the inflammatory response, has been demonstrated [209]. The intensification of inflammation noted in individuals with PMF could be linked to inadequate intake of fiber and short-chain fatty acids [210]. Mechanistically, fiber and unsaturated lipids within the Mediterranean diet undergo fermentation by gut bacteria, yielding metabolites that exhibit anti-inflammatory properties [211]. Nevertheless, additional investigations are needed to ascertain whether the Mediterranean diet can serve as a strategic approach to foster health by modulating the intestinal microbiome and alleviating inflammation in PMF.

## 7. Conclusion

The precise etiopathogenic mechanisms underpinning the development and progression of PMF still remain incompletely understood. Unfortunately, existing therapeutic strategies remain inadequate in halting disease progression or precluding its transformation into leukemia.

Inflammation, a pivotal and recurrent hallmark throughout the evolution of PMF, has emerged as a highly promising target for clinical intervention. The overproduction of cytokines, the presence of ROS, and immune dysregulation engage in a pernicious cycle, culminating in genetic instability, disease progression, and immune evasion. These factors further transmit signals to act upon BM stromal cells, thereby instigating a cascade of events that ultimately lead to BM fibrosis. The intricate interplay among these elements has been comprehensively documented in the scientific literature.

An integrated therapeutic paradigm that combines anti-inflammatory regimens with established JAK inhibitors holds substantial promise in disrupting the inflammatory cascades and the complex interactions between HSPCs and stromal cells within the hematopoietic niche. This approach thus offers a more efficacious treatment strategy, potentially revolutionizing the management of PMF.

The clinical manifestations of PMF are highly heterogeneous, typically characterized by cytopenia, extramedullary hematopoiesis, hepatosplenomegaly, and cytokine-mediated systemic symptoms. Further clinical investigations are indispensable for elucidating the relationship between biological characteristics and inflammation, as well as for identifying those patients who are most likely to derive substantial benefits from anti-inflammatory therapies.

Looking toward the future, translational clinical trials will play an instrumental role in determining the optimal targeting of these pathways in PMF patients. The overarching goal is to enhance long-term outcomes, improve survival rates, and potentially achieve a cure, thereby bringing us closer to effectively managing this complex and challenging disorder.

## Figures and Tables

**Table 1:** List of drugs ongoing clinical trials in myelofibrosis.

Therapeutic strategy	Mechanism	NCT number	Agent and clinical design	Phase	Status
JAKi	Type II inhibitor of JAK2	NCT06343805	AJ1-11,095 for MF patients who failed Type I JAK2 inhibitors	Phase 1	Recruiting
	Pseudokinase (JH2)-targeting	NCT06313593	INCB160058 for patients with MPN	Phase 1	Recruiting
	JAK2/FLT3 inhibitor	NCT05153343	Flunotinib for patients with MPN	Phase 1/2	Recruiting
	Targeting nonreceptor Janus kinases	NCT05279001	Jaktinib for R/R MF patients to JAKi	Phase 1	Recruiting
	JAK/ROCK inhibitor	NCT06245941	TQ05105 combined with BCL-2 inhibitor TQB3909	Phase1/2	Recruiting

Immunomodulatory therapy	CXCR4 enriched T regs cell	NCT05423691	CK0804 as addition to ruxolitinib	Phase 1	Recruiting
	Anti-CALRmut/anti-CD3 bispecific antibody	NCT06150157	JNJ-88549968 for CALR-mutated MPN	Phase 1	Recruiting
	Mutant CALR-peptide-based vaccine	NCT05025488	Vaccination with a peptide derived from the CALR exon 9 mutations in patients with CALR-mutated MPN	Phase 1	Recruiting
	Neoantigen vaccine regimen, CTLA-4 antibody	NCT05444530	VAC85135 with ipilimumab for MPN	Phase 1	Active, not recruiting
	Anti-hemojuvelin monoclonal antibody	NCT05320198	DISC-0974 for patients with MF or myelodysplastic syndromes and anemia	Phase 1/2	Recruiting

Anticytokine therapy	TGF-β ligand traps	NCT05037760	KER-050 mono or in combination with ruxolitinib	Phase 2	Recruiting
	CXCR1/CXCR2 inhibitor	NCT05835466	Reparixin for MF after prior treatment, and JAKi–ineligible/refusers	Phase 2	Recruiting

NF-*κ*B signaling inhibitor	BET proteins inhibitor	NCT02158858	CPI-0610 mono or in combination with ruxolitinib	Phase 2	Active, not recruiting
		NCT04279847	INCB057643 in MF and advanced myeloid neoplasms	Phase 1	Recruiting

Small molecule inhibitor	BTK inhibitor	NCT04655118	TL-895 for R/R or JAKi–intolerant MF	Phase 2	Recruiting
		NCT05280509	TL-895 with ruxolitinib for JAKi–naïve MF and suboptimal ruxolitinib responders	Phase 1/2	Recruiting
	Mechanism	NCT number	Agent and clinical design	Phase	Status
	MDM2 inhibitor	NCT06479135	Navtemadlin add-on to ruxolitinib for JAKi-naïve MF patients who have a suboptimal response to ruxolitinib	Phase 3	Recruiting
		NCT03662126	Navtemadlin vs. BAT for R/R MF	Phase 2/3	Recruiting
		NCT04878003	Navtemadlin or TL-895 for JAKi–naïve MF	Phase 2	Recruiting
		NCT04640532	Navtemadlin in combination with TL-895 for R/R MF	Phase 2	Recruiting
			Navtemadlin for the JAKi–intolerant MF		
	LSD1 inhibitor	NCT06351631	Bomedemstat mono for MPN	Phase 3	Recruiting
		NCT05569538	Bomedemstat in combination with ruxolitinib for MF	Phase 2	Recruiting
	CDK 8/19 inhibitor	NCT06397313	RVU120 mono or in combination with ruxolitinib	Phase 2	Recruiting
	CDK4/6 inhibitor	NCT05714072	Abemaciclib in combination with ruxolitinib	Phase 1	Recruiting
	PIM1 inhibitor	NCT04176198	Nuvisertib	Phase 1/2	Recruiting
	S100A8/A9 signaling inhibitor	NCT06327100	Tasquinimod mono or in combination with ruxolitinib	Phase 2	Recruiting

Protein degrader	CK1α degraders	NCT06378437	GLB-001 in patients with myeloid malignancies	Phase 1	Recruiting

Enzyme inhibitor	PARP inhibitor	NCT06218628	Talazoparib in combination with pacritinib in patients with MPN unresponsive to JAK2 inhibitors	Phase 1	Recruiting
	Telomerase inhibitor	NCT04576156	Imetelstat vs. BAT for suboptimal ruxolitinib responders	Phase 3	Recruiting
			Imetelstat in combination with ruxolitinib		
		NCT05371964	Imetelstat in combination with ruxolitinib	Phase 1	Recruiting
	Selective inhibitor of nuclear export	NCT04562389	Selinexor in combination with ruxolitinib for JAKi-naïve patients	Phase 3	Recruiting
		NCT05980806	Selinexor mono for patients with JAKi–naïve MF and moderate thrombocytopenia	Phase 2	Recruiting
	The hypomethylating agent	NCT01787487	Azacytidine in combination with ruxolitinib	Phase 2	Recruiting
	Liposome-encapsulated daunorubicin–cytarabine	NCT03878199	CPX-351 in combination with ruxolitinib for advanced phase MPN	Phase 1/2	Recruiting

Abbreviations: BAT, best available therapy; JAKi, Janus kinase inhibitor; MF, myelofibrosis; mono, monotherapy; MPN, myeloproliferative neoplasm; R/R, relapsed/refractory.

## Data Availability

No datasets were generated or analyzed during the current study.
